# Identification and Characterisation of Heat Shock Protein 70 in Thermal Stressed *Blastocystis* sp

**DOI:** 10.1371/journal.pone.0095608

**Published:** 2014-09-02

**Authors:** T. Gaythri, K. Suresh, B. Subha, R. Kalyani

**Affiliations:** 1 Department of Parasitology, University of Malaya, Kuala Lumpur, Malaysia; 2 Department of Genetics and Molecular Biology, University of Malaya, Kuala Lumpur, Malaysia; California State University Fullerton, United States of America

## Abstract

Protistan parasites in order to ensure their viability and demonstrate successful progression in their life cycle need to respond towards various environmental stressors. *Blastocystis* sp. is known to be the most commonly found intestinal protistan parasite in any human stool surveys and has been incriminated to be responsible for diarrhea and bloating stomach. The present study demonstrates for the first time the presence of HSP70 in subtypes of *Blastocystis* sp. when the cultures were subjected to temperature of 39 and 41°C where the growth of parasites was reduced to a minimum to majority being granular forms. The growth of parasites exposed to higher temperatures however doubled compared to the controls when the parasites were re-cultured back at 37°C. Upon thermal stress at 41°C, subtype 3 and subtype 5 isolates' growth reached up to 2.97×10^6^ and 3.05×10^6^ cells/ml compared to their respective controlled culture tubes at 37°C which peaked only at 1.34×10^6^ and 1.70×10^6^ cells/ml respectively. The designed primer set that amplified *Blastocystis* sp. subtype 7 HSP70 gene in subtypes 1, 3 and 5 was against a conserved region. The gene was amplified at 318 bp. The multiple sequence alignment showed that the targeted sequence length ranges from 291–295 bp. The pair wise alignment result showed that the sequence identity among the four sequence ranges from 88% to 96%. These findings were further evidenced by the up regulation of HSP70 gene in thermal stressed isolates of subtype 3 and 5 at 41°C. Higher number of granular forms was significantly found in thermal stressed isolates of subtype 3 and 5 which implicates that this life cycle stage has a role in responding to thermal stress.

## Introduction

*Blastocystis* sp. is reported to be the most commonly found organism in any stool surveys. The prevalence in developing and developed nations is 30–50% and 1.5–10% respectively [1]. It has been previously reported that symptomatic and asymptomatic isolates show different phenotypic and genotypic characteristics [2] When clinical symptoms are expressed it is natural that infected persons will seek medical help but surviving asymptomatic forms will continue to live probably as an unnoticed commensal.

The body is subjected to various stresses much of which is contributed to the current lifestyles. The vulnerability of humans to various other bacteria and virus infection especially caught during travels as well as from air and water borne transmission cannot be underestimated. In the tropics, the increasing incidences of vector borne diseases such as dengue and malaria can increase body temperature up to even 41°C.

The question as to what would happen to a *Blastocystis* infected person subjected to temperature increase due to other infections has never been investigated. Protistan parasites in order for survival must obviously respond to such environmental stressors to ensure their viability and successful survival. Studies previously have shown oxidative-stressed *Plasmodium falciparum* showed increased co-ordination and stage-dependant expression of its antioxidant enzymes and heat shock proteins [3]. This parasite also slows its metabolism and progresses through its developmental cycle at a reduced level by entering into a hibernatory state as a respond to isoleucine (amino acid) starvation [4].

Heat shock protein (HSP) 70 has been identified as a molecular chaperone that gets expressed in response to thermal stress. To date although the sequence of *Blastocystis* sp. subtype 7 heat shock protein 70 (HSP70) is available in the GenBank [5], there has been no further research carried out on this gene. Thus, the present study is aimed in identifying the presence of HSP70 in subtypes of *Blastocystis* sp. and understanding its role in *Blastocystis* sp. when thermal stress is induced.

## Results

The growth of thermal stressed *Blastocystis* sp. isolates were observed when re-cultured at 37°C. Isolates of *Blastocystis* sp. subtypes 1, 3, and 5 in Jones's medium containing 10% horse serum were thermal stressed for 24 hours at 39°C and 41°C respectively. Parasites were enumerated after 24 hours of thermal stress. All the isolates were incubated back at controlled temperature (37°C) and maintained for 13days. Parasites were enumerated once every 3 days up to day 13 during when 1 ml of supernatant from each culture tube was replaced respectively with 1 ml of fresh Jones' medium containing 10% horse serum. This step is important to ensure that the source of stress was only temperature and not due to depleting nutrients in the culture medium.

**Figure 1:**
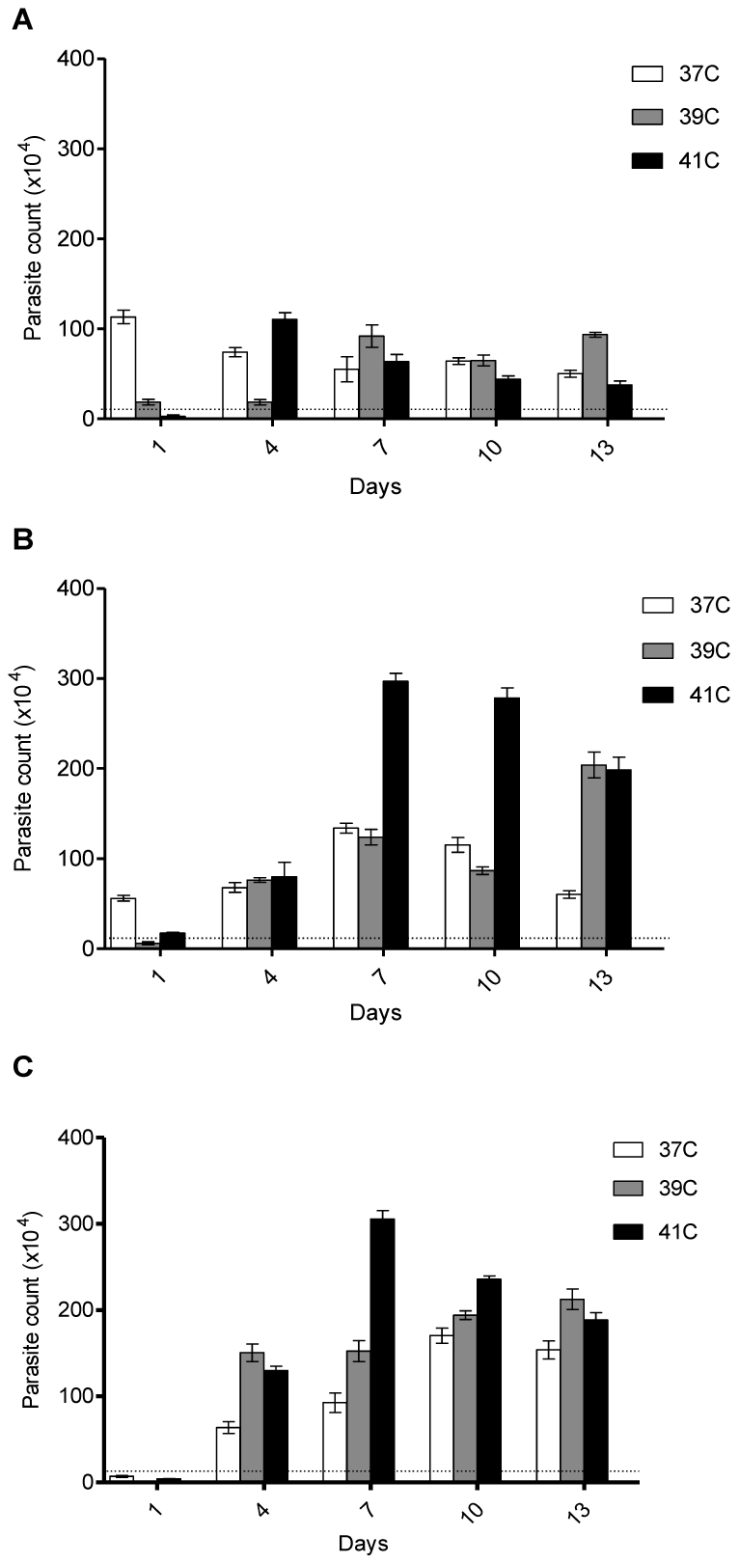
Growth of *Blastocystis* sp. Controlled temperature (37°C) and thermal stress temperatures (39°C and 41°C) were used. (A) Subtype 1 did not show significant growth difference between control and thermal stressed isolates. (B) Subtype 3 and (C) Subtype 5 which thermal stressed at 41°C showed significantly higher growth compared to isolate at 37°C.

All the three subtypes exposed to three different temperatures showed peak growth on day 7. Among the three temperatures the optimal growth of subtype 1 was at 37°C, reaching 1.13×10^6^ cells/ml compared to the initial inoculation which was 0.1×10^6^ cells/ml (Figure 1A).

However, *Blastocystis* sp. subtype 3 and 5 isolates showed an increased growth when parasites were thermal stressed at 41°C. After 24 hours incubation at 41°C, the parasite count of both subtypes reduced tremendously 0.02×10^6^ and 0.04×10^6^ cells/ml respectively for subtype 3 and 5 isolates (Figure 1B and 1C). However, when the thermal stressed subtype 3 and 5 samples was re-cultured at 37°C, peak parasite count observed at 2.97×10^6^ and 3.05×10^6^cells/ml respectively. Thermal stressed subtype 3 and 5 isolates showed the highest growth at 41°C (p<0.001, Bonferroni test), whereas for subtype 1, the highest parasite count was seen at 37°C. This proved that there is an interaction between subtypes and temperature.

As the thermal stressed subtype 3 and 5 isolates showed significantly higher growth compared to their respective control cultures, another similar experiment was carried out to identify the growth of thermal stressed *Blastocystis* sp. based on number of vacuolar and granular forms. Similar to previous experiment, isolates of *Blastocystis* sp. subtype 1, 3, and 5 in Jones's medium containing 10% horse serum were thermal stressed for 24 hours at 41°C. Controlled isolates were continuously maintained at 37°C. Parasites were enumerated based on their morphology after 24 hours of thermal stress. All the isolates were incubated back at controlled temperature (37°C) and maintained for 10 days. Parasites were enumerated every 3 days once up to day 10. Upon enumeration, 1 ml of fresh Jones' medium containing 10% horse serum will be replaced with 1 ml of supernatant for each isolate to make sure the nutrient is at constant level.

**Figure 2:**
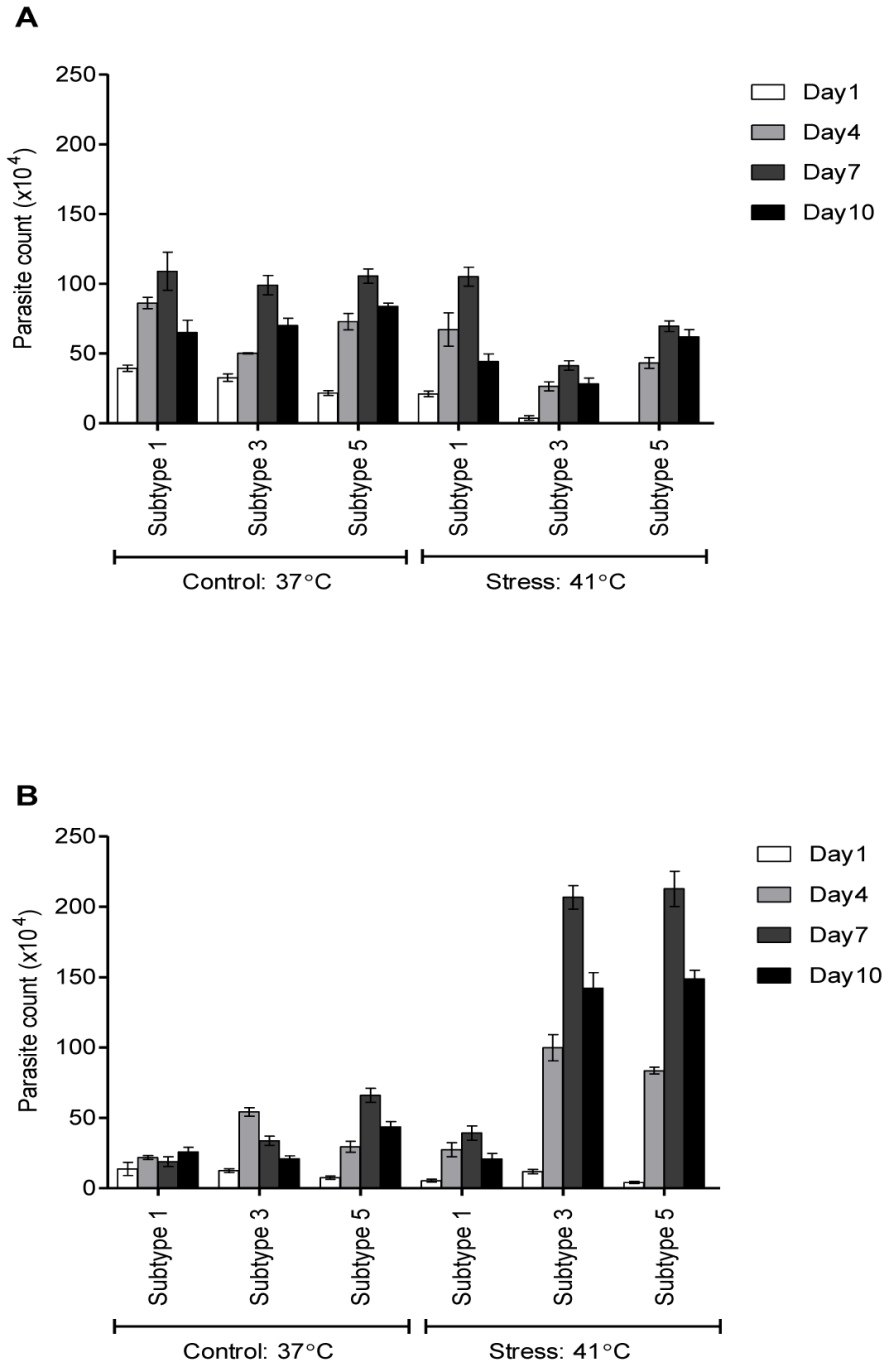
*Blastocystis* sp. forms in control and thermal stressed isolates. Isolates of subtype 1, 3, and 5 were grown at controlled temperature (37°C) and thermal stressed temperature (41°C). (A) Vacuolar form was highly significant in controlled isolates compared to their respective stressed isolates of all the three subtypes. (B) Granular form was highly significant in the thermal stressed isolates of subtype 3 and 5.

In this experiment it was shown that growth of subtypes appears to be temperature sensitive. A higher number of vacuolar forms were seen in the control cultures grown at 37°C (Figure 2A). Controlled isolates of subtype 1, 3 and 5 showed peaked parasite count of vacuolar form on day 7 reaching 1.09×10^6^, 0.99×10^6^ and 1.05×10^6^ cells/ml respectively. Number of granular forms of subtype 3 and 5 isolates reached 2.07×10^6^ and 2.13×10^6^ cells/ml respectively (Figure 2B). However, for subtype 1, the number of granular forms was not significantly seen when exposed to thermal stress.

**Figure 3:**
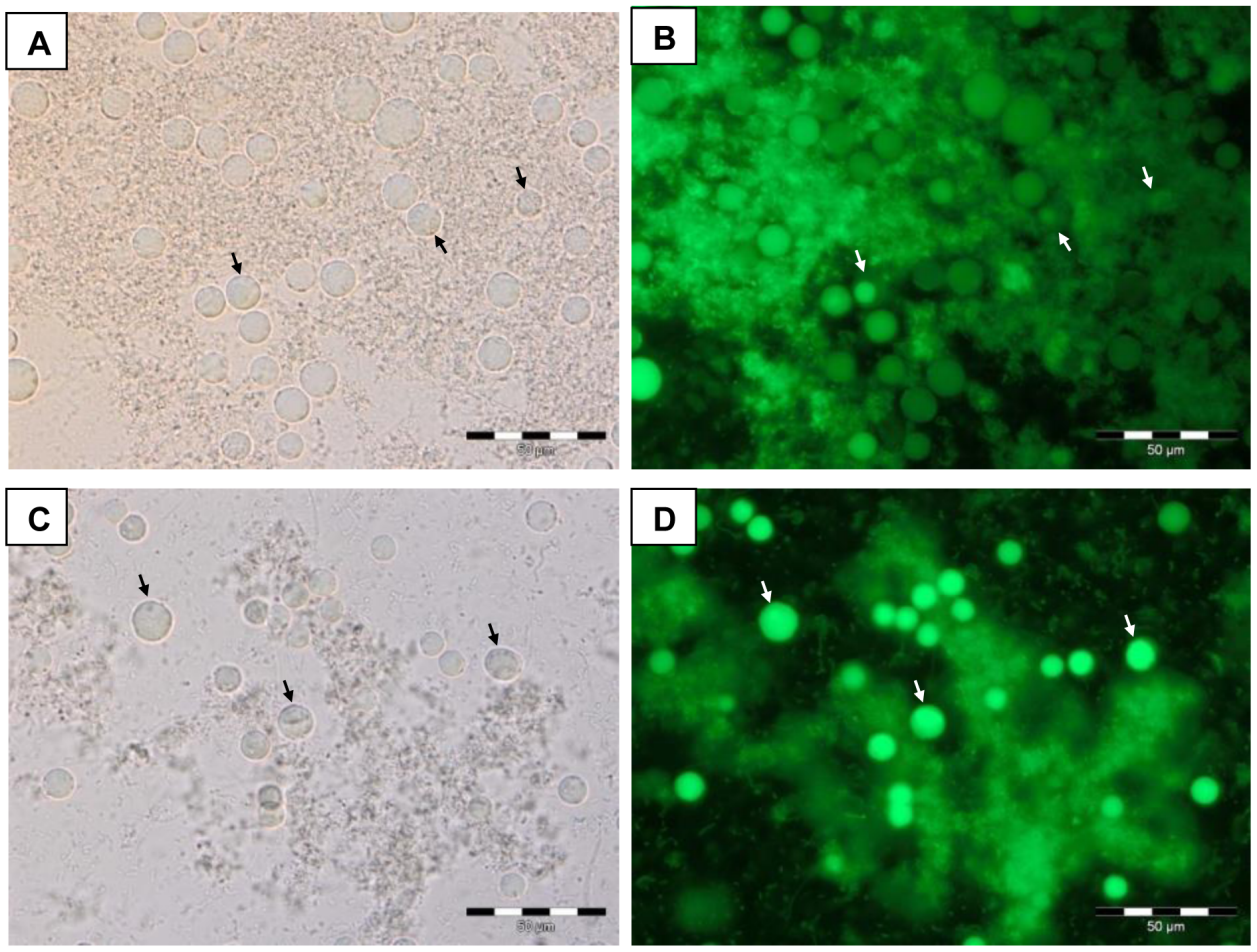
Bright field and epifluorescence images of *Blastocystis* sp. stained with acridine orange (×400). Image A and B is the control isolate at 37°C, while C and D is the thermal stressed isolate at 41°C. Note: The vacuolar forms in control isolates are stained dull green (Image B), and the granular form (arrow) in thermal stressed isolates are stained bright green (Image D).

Acridine orange stain was used to elucidate the biochemical difference between vacuolar and granular forms. *Blastocystis* sp. culture was incubated at 37°C and 41°C for 24 hours. Following the 24 hours incubation, one drop of culture sediment was mixed with acridine orange and observed under fluorescence microscope (magnification ×400). Under the bright field, control culture had higher parasite count after 24 hours incubation with a mix of vacuolar and granular form (Figure 3A). The number of parasite was lesser in thermal stressed culture and more granular form was observed (Figure 3C). The respective epifluorescence image showed that the vacuolar form was stained dull green while the granular form was stained bright green (Figure 3B and 3D). However, the green fluorescent intensity of the granular form in thermal stressed culture is higher compared to the control culture.

**Figure 4:**
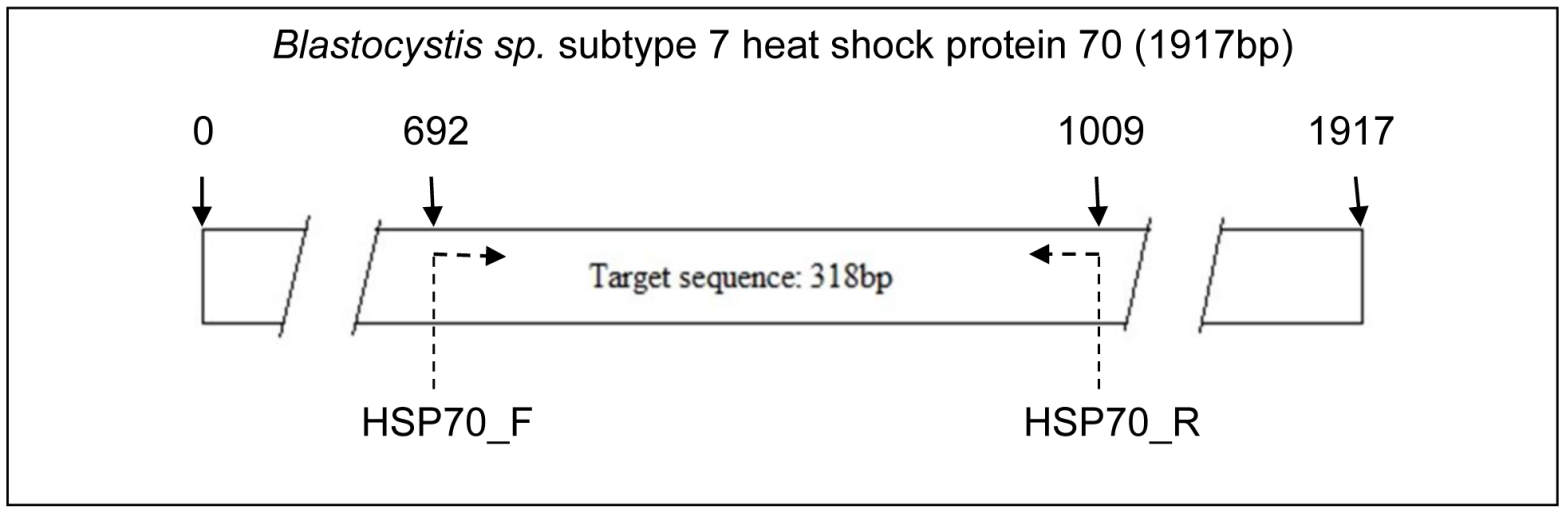
Localization of the target sequence in the *Blastocystis sp.* heat shock protein 70 (HSP70) gene for subtype 7.

The sequence of *Blastocystis* sp. subtype 7 heat shock protein 70 (HSP70) gene was obtained from EMBL GenBank. The length of *Blastocystis* sp. HSP70 gene sequence is 1917 bp with 638 amino acids. NCBI Primer Blast online tool was used to design a pair of primers which was able to target the conserved region of this gene. One pair of primers was chosen out of five primer pair based on the melting temperature, percentage of GC content and self complementarities of the primers. This primer targets the region on the sequence which starts from 692 bp to 1009 bp with the targeted sequence length of 318 bp (Figure 4). This targeted sequence was blast using the NCBI blastp online tool to determine the conserved region. It was identified as the nucleotide binding domain (NBD) _sugar-kinase_HSP70_actin super family.

**Figure 5:**
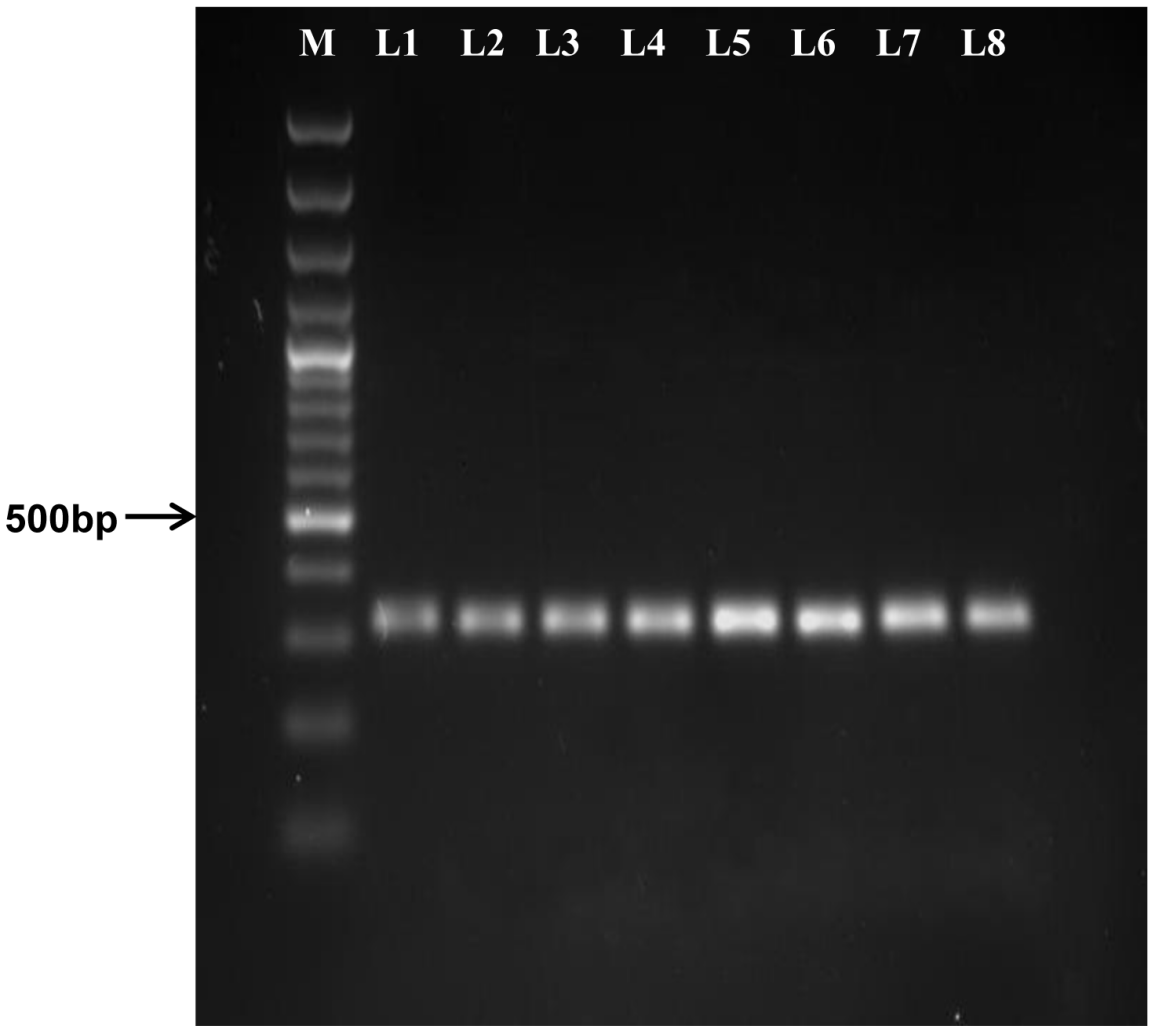
Agarose gel image of the amplified *Blastocystis sp* heat shock protein (HSP) 70 gene. The amplicon size of the target region is 318 bp. Lane M = DNA markers of 100 bp plus DNA ladder (Fermentas, USA); lane 1–4 =  subtype 1; lane 5–7 =  subtype 3 and lane 8 =  subtype 5.

The designed primer set was used to amplify the *Blastocystis* sp. subtype 7 HSP70 gene. Since the primer was designed against a conserved region, it was able to amplify the HSP70 gene in subtype 1, 3, and 5 of *Blastocystis* sp. The gene was amplified at 318 bp as targeted (Figure 5). The targeted bands were excised from agarose gel and purified using gel extraction kit. Three samples were randomly chosen and sequenced.

**Figure 6:**
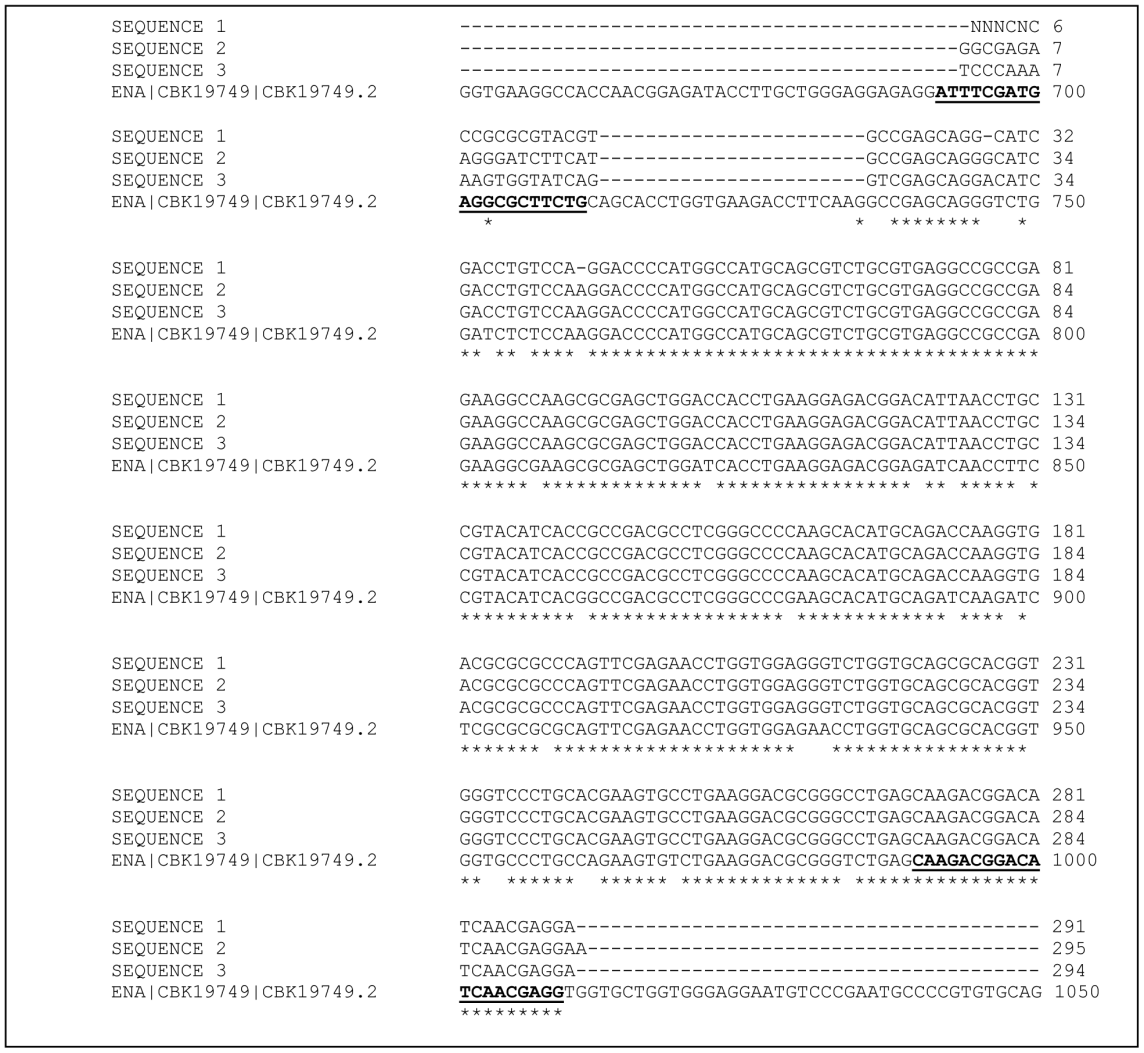
ClustalW2 multiple sequence alignment of the target sequence *Blastocystis sp.* heat shock protein 70. Three random isolates of *Blastocystis sp*. was used. Primers (HSP70_F and HSP70_R) are indicated in bold and underlined.

The DNA sequencing results were aligned against the *Blastocystis* sp. subtype 7 HSP70 gene using the ClustalW2 online software. The multiple sequence alignment showed that the targeted sequence length ranges from 291–295 bp (Figure 6). The pair wise alignment result showed that the sequence identity among the four sequence ranges from 88% to 96%.

**Figure 7:**
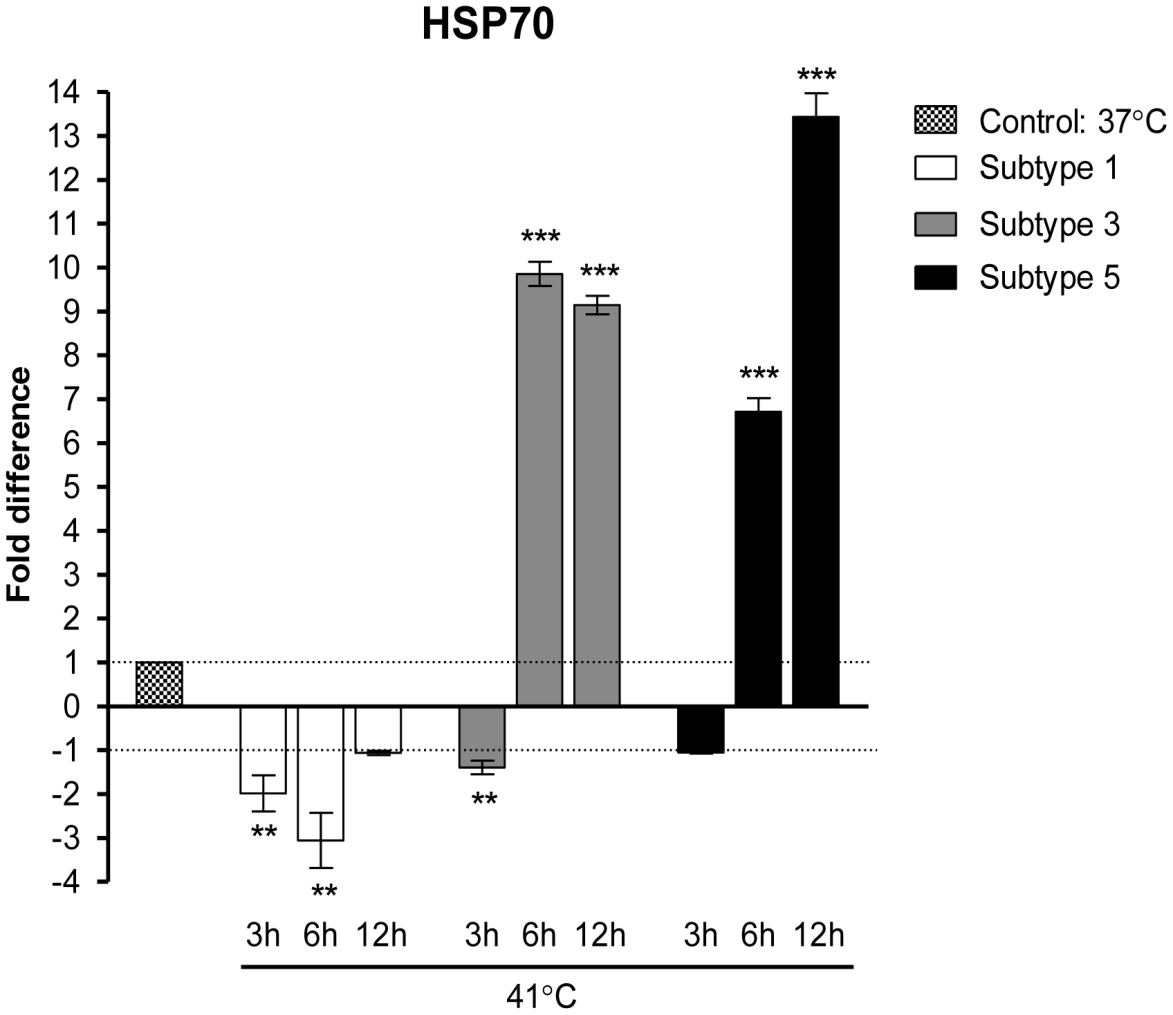
Gene expression profile of HSP70 in thermal stressed *Blastocystis sp*. Isolates of subtype 1, 3, and 5 were thermal stressed at 41°C for three different lengths of time. Values are presented in fold difference observed in comparison with control (37°C) and normalized against endogenous gene (ssu_rRNA). A positive fold difference refers to up regulation and vice versa. Student t-test was performed and a statistically significant difference was found between control and thermal stressed samples (**p<0.01, and ***p<0.001).

The targeted sequence was then used to customize the primer for real-time PCR. The Custom Taqman Gene Expression assay showed that the HSP70 gene was down regulated in subtype 1 at all the time points of 3, 6, and 12 hours of thermal stress at 41°C (Figure 7). A similar pattern was observed at 3 h incubation for subtypes 3 and 5. However, the gene was significantly up regulated at 6 and 12 h incubation at 41°C for both subtypes 3 and 5 (p<0.001, Student t-test). Real-time PCR result shows that there is a correlation between the up regulation of HSP70 and the significant growth of subtypes 3 and 5 (Figure 1B and 1C) after incubation at 41°C can be correlated to the up regulation of HSP70 as showed by the fold difference at 6 and 12 h incubation. Whereas, subtype 1 has no significant effects towards thermal stress (Figure 1A) which can be evidenced by the down regulation of HSP70 during the 3, 6 and 12 h incubation at 41°C. Thus, *Blastocystis* sp. responds to stressful environments for survival by HSP70 gene regulation.

## Discussion

One of the most important aspects of parasite survival is its ability to respond in stressful environments. Mainly, there are four major cellular stresses encountered by protistan parasites namely thermal, nutritional deprivation, osmotic and oxidative stress. The study on the oxidative stress response was previously done to evaluate the susceptibility of metronidazole resistant strain of *Blastocystis* sp. to nitrosative stress [6]. *Blastocystis* sp. was found to be susceptible to nitric oxide (NO) by showing the sign of necrosis and apoptosis like cell death. However, the present study was able to suggest that *Blastocystis* sp. has developed a survival mechanism against NO by down regulating epithelial inducible nitric oxide synthase (iNOS) expression and inhibit epithelial NO production. This phenomenon was also reported in parasites such as *Entamoeba* [7] and *Giardia* [8] and bacteria like *H.pylori* [9].

*Giardia lamblia* in response to an increase of temperature showed a reduction of normal protein synthesis and an increase heat shock proteins (HSPs) synthesis [10]. The observation concurs also with *Blastocystis* sp. in that the parasite numbers decreased to a minimal level with a significant presence of HSP70 when exposed to thermal stress. The present study showed that *Blastocystis* sp. when thermally stressed synthesizes heat shock proteins (HSPs) to cope with the thermal stress. The observation concurs with a previous study in that the metabolism of stress proteins can be affected when exposed to stressful environment as evidenced by the increase in HSP70 level which correlated with reduced fitness of an organism [11]. In Drosophila, cells which continuously expressing HSP70 showed reduced growth compared to the controlled cells, however it reverted to normal growth once HSP70 was removed from the cytoplasm [12]. Another study showed that this organism increased copy number of HSP70 upon heat stress which eventually led to a defect in the development of the fly [13]. Highest growth performance was achieved for both *Abudefduf vaigiensis* [14] and *S. sarba* [15] when expression of HSP70 was lowered. Another study proved that *E. sennetsu* an intracellular bacteria showed larger induction of HSP70 due to the temperature shift (37°C to 41°C) during transfer from vector temperature to human body temperature [16]. The higher induction of stress protein is important when this organism infects a human host.

Sorensen et al [11] reasoned out that higher expression of HSPs diverts energy meant for growth development and reproduction towards creating a protection buffer from stressful environment. This could be the likely reason why the growth rate was reduced in *Blastocystis* sp. when exposed to thermal stress and doubled when re-cultured at normal temperature.

**Figure 8:**
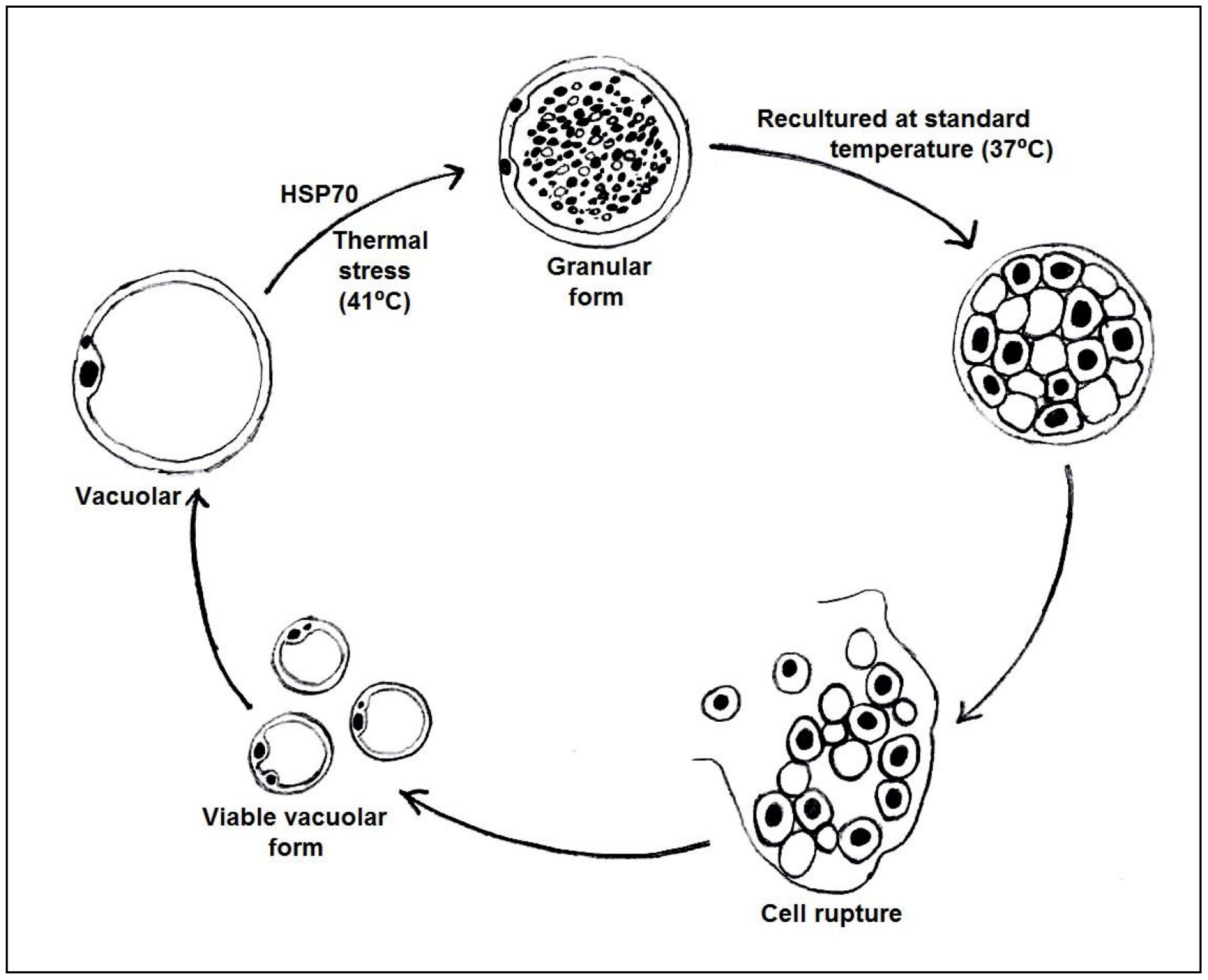
Biological illustration of thermal stressed *Blastocystis sp*.

The interesting point to observe that majority of stressed organisms were the granular forms which implies that the doubling effect in growth when re-cultured at 37°C is probably due to the granules being released to form the viable vacuolar forms. This postulation is illustrated in Figure 8. Binary fission is the only currently accepted plausible mode of reproduction. However this would not account for the high number of vacuolar forms seen within a short time providing evidence for a role for the granular forms. We have previously highlighted this fact [17] but were refuted [18]. The present experiment elegantly confirms our earlier suspicion as there is no other rationale to justify the large numbers seen within a short time. Studies have previously reported that culture conditions including increased serum concentration, axenization, antibiotics, and use of different culture media does trigger granular production [1]. The formation of granules in this case could also be due to a stress response. Previous studies have proposed three types of granular form of *Blastocystis* sp. as metabolic, reproductive, [19] and lipid granules [20]. The reproductive type has been suggested to have the ability to develop into new daughter cells [19].

This postulation was further confirmed by the acridine orange staining method. The green fluorescence were more intense in organisms from thermal stressed cultures than the control ones implying that there were more granular forms as shown previously [21].

A targeted HSP70 gene sequence of 318 bp proved to be present in all the isolates of three subtypes (Figure 5). Further analysis on the targeted region showed that it was conserved for sugar-kinases of HSP70 actin superfamily. Actin has been known to contribute to cellular processes such as metabolism, growth and apoptosis. Heat shock proteins have been attributed to pathogenesis of diseases [22]. The present findings can be extrapolated to draw possible implication. A dengue patient infected also with *Blastocystis* sp. can have fever peaks which can enable the parasites to be thermally stressed and thereby reduce the parasite count to a minimum. When the patient recover and regains his normal temperature perhaps after days of illness, *Blastocystis* then rise to higher numbers.

The present study is the first to demonstrate HSP70 in thermal stressed *Blastocystis* sp. This important finding especially in terms of the parasites ability to multiply at a higher rate after exposure to high temperature is an important one to explain the gastrointestinal symptoms of patients infected with *Blastocystis* suffering from malaria and dengue. The findings of granular forms as a larger proportion of thermal stressed *Blastocystis* implies that these forms are triggered to increase progeny forms for the survival of the organism.

## Materials and Methods

### 1.1. Source of the *Blastocystis* sp. Isolate

*Blastocystis* sp. isolates used in this experiment were isolated from stool samples received at the Department of Parasitology, Faculty of Medicine, University of Malaya. Collection and storage of samples for research purposes was approved by University of Malaya Ethics committee. All samples were de-indentified prior to this study, and none of the authors had access to personal information associated with the donors. The stool samples were collected from members of the aborigine committee in Kuala Langat a district in the outskirts of Kuala Lumpur. Out of 30 stool samples collected, 8 were found to be positive for *Blastocystis sp*.

### 1.2. In vitro cultivation of *Blastocystis sp*. Isolates

*Blastocystis sp*. was isolated from the stool samples of school children and local communities by in vitro cultivation using Jones' medium [23] supplemented with 10% horse serum and incubated at 37°C [24], [25], [26]. In vitro cultures were done by inoculating approximately 50 mg of stool sample in 3 ml of Jones' medium. Isolated parasites were maintained by sub-culturing once every 3 to 4 days in Jones' medium for at least 1 month prior to the phenotypic analysis.

### 1.3. Genomic DNA Preparation

*Blastocystis* sp. isolates grown in Jones' medium were harvested by centrifugation at 1000 g for 5 min and washed twice using sterile phosphate buffered saline (PBS) (pH 7.4). The harvested parasites were used to purify its genomic DNA. The QIAamp DNA Stool Mini Kit (Qiagen, Australia) was used to extract the DNA according to the manufacturer's protocol. The concentration and purity of DNA was measured using Nanodrop 2000 (Thermo Scientific, USA).

### 1.4. Subtyping of *Blastocystis* sp

The genomic DNA of all the eight isolates of *Blastocystis* sp. were amplified by polymerase chain reaction (PCR) using seven sets of sequenced-tagged site (STS) primers (Yoshikawa, 2003). Amplification of 2 µl genomic DNA was carried out in 20 µl reaction containing 2.5 µl of 10X Taq Buffer with KCL, 3.125 mM of MgCl_2_, 0.5 mM of deoxyribonucleotide triphosphates, 0.25 mM of forward and reverse primer and 1 U Taq DNA polymerase (recombinant) (Fermentas, USA). The thermal profile was programmed with one cycle of initial denaturation at 95°C for 5 min; followed by 30 cycles of denaturation at 95°C for 1 min, annealing at 56.3°C for 1 min 30 sec and extension at 72°C for 1 min; one cycle of final extension at 72°C for 10 min and final infinite holding at 10°C (Thermal Cycler Bio-rad, USA). The amplified products were examined by electrophoresis using 1.5% agarose gels (Pronadisa, Spain) in Tris-Borate-EDTA buffer and stained with ethidium bromide. Gels were visualized and photographed using ultra-violet gel documentation system (UVP, Germany).

### 1.5. Analysis on thermal stressed *Blastocystis* sp. growth

The parasites of each isolates were pooled together from day 3 cultures to make a final concentration of 1×10^5^ cells/ml in 3 ml Jones' medium supplemented with 10% horse serum. Thermal stress was introduced by incubating two sets of each isolate at 39°C and 41°C respectively. After 24 hours stressing the parasites at these temperatures, the parasites from all the culture tubes were re-cultured at 37°C to resume optimal growth condition and this was continuously maintained for 13 days. One set which consist of 3 culture tubes of each isolate was continuously incubated at 37°C which was used as the control. Each set was prepared in triplicate and all the cultures were kept in airtight tubes. Parasite count was done after 24 hours of heat exposure and at every 3 days interval for up to 13 days. This was done using haemocytometer chamber (Improved Neubauer, Hausser Scientific) with 0.5% Trypan blue solution as viability indicator. Only viable cells which did not take up Trypan blue stain were counted. Statistical analyses were carried out using SPSS version 20.

### 1.6. Analysis on the thermal stressed *Blastocystis* sp. forms

The parasites of each isolate were pooled together from day 3 cultures to make a final concentration of 1×10^5^ cells/ml in 3 ml Jones' medium supplemented with 10% horse serum. Thermal stress was introduced by incubating one sets of each isolate at 41°C. After 24 hours of thermal stress, all the tubes were re-cultured at 37°C to resume optimal growth condition and maintained for 10 days. One set of each isolate was continuously incubated at 37°C which was used as the control. Each set was prepared in triplicate and all the cultures were kept in airtight tubes. Number of vacuolar and granular forms of *Blastocystis* sp. was calculated after 24 hours of heat exposure and at every 3 days interval for up to 10 days. Granular and vacuolar forms were differentiated by seeing through the haemocytometer chamber (Improved Neubauer, Hausser Scientific) with 0.5% Trypan blue solution as viability indicator. Only viable cells which did not take up Trypan blue stain were counted. Statistical analyses were carried out using SPSS version 20.

### 1.7. Acridine orange staining of thermal stressed *Blastocystis* sp

The isolates at control temperature 37°C and thermal stressed for 24 hours were stained with acridine orange solution. A drop of culture sediment containing parasites was mixed with a drop of acridine orange (0.01 mg/ml) on a clean glass slide. The prepared slide was viewed with a fluorescence microscope (Olympus, Japan).

### 1.8. RNA extraction of *Blastocystis* sp. and reverse transcription to cDNA

All the isolates used for viability test were subjected to RNA extraction. Approximately 1×10^6^ parasites from each isolate were washed with phosphate buffer saline (PBS) (Sigma-Aldrich, USA). RNA Extraction was carried out using TRIzol reagent (Life Technology USA) according to manufacturer's instruction instructions. The concentration and purity of RNA was measured using Nanodrop 2000 (Thermo Scientific, USA). 50 ng/µl RNA was reverse transcribed in 20 µl reaction using the ImProm-II Reverse Transcription System (Promega, USA) as per the manufacturer's protocol.

### 1.9. Primer designing and amplification *Blastocystis* sp. heat shock protein 70 (HSP70) gene

The sequence of *Blastocystis* sp. subtype 7 heat shock protein 70 (ENA|CBK19749.2) was obtained from UniProtKB/TrEMBL online database. Primers for this sequence were specifically designed using the NCBI Primer Blast online tool. A pair of HSP70 primer of forward (HSP70_F): 5′-ATTTCGATGAGGCGCTTCTG-3′ and reverse (HSP70_R): 5′-CCTCGTTGATGTCCGTCTTG-3′ (data not published). The cDNA of all the isolates of *Blastocystis* sp. were amplified by polymerase chain reaction (PCR) using the pre-designed HSP70 primer set. Amplification of 1 µl cDNA was carried out in 20 µl reaction containing 2.5 µl of 10X Taq Buffer with KCL, 4.0 mM of MgCl_2_, 0.5 mM of deoxyribonucleotide triphosphates, 0.25 mM of forward and reverse primer and 1 U Taq DNA polymerase (recombinant) (Fermentas, USA). The PCR conditions consisted of one cycle of initial denaturation at 94°C for 5 min; followed by 35 cycles of denaturation at 94°C for 30 sec, annealing at 63.0°C for 1 min and extension at 72°C for 3 min; one cycle of final extension at 72°C for 10 min and final infinite holding at 4°C (Thermal Cycler Bio-rad, USA). The amplified products were examined by electrophoresis using 1.5% agarose gels (Pronadisa, Spain) in Tris-Borate-EDTA buffer and stained with ethidium bromide. Gels were visualized and photographed using ultra-violet gel documentation system (UVP, Germany).

### 1.10. Sequencing

The targeted bands in the agarose gel were excised out and the gel was extracted using the QIAquick Gel Extraction Kit (Qiagen, Australia). Three purified products with highest concentration were chosen and sent to First BASE Laboratories for DNA sequencing service. All the three sequencing results were aligned against the *Blastocystis* sp. subtype 7 HSP70 gene using the ClustalW2 software to determine the targeted region.

### 1.11. Real-time polymerase chain reaction analysis

Real-time PCR analysis was carried out to determine the HSP70 gene expression of thermal stressed *Blastocystis* sp. Isolates of subtype 1, 3, and 5 were incubated at 41°C for three length of time (3, 6, and 12 h). Isolate which was maintained at optimal conditions was used as the control. Complementary DNA was extracted from the control and thermal stressed samples and real-time PCR was performed using the Custom Taqman Gene Expression Assay (Applied Biosystems) with the Applied Biosystems StepOne System. The threshold cycle (C_T_) value of each sample was measured and compared to endogenous gene, ssu_rRNA. Fold changes were measured using the ΔΔC_T_ method. Relative transcripts were determined by the formula 2^−ΔΔC^_T_.
